# Genealogies of organizational closure: from logical paradox to mind in life

**DOI:** 10.3389/frai.2026.1806435

**Published:** 2026-08-27

**Authors:** Sergio Rubin, Andrea Roli, Pasquale Stano, Luisa Damiano

**Affiliations:** 1 Research Center for Complex Systems (CRiSiCo), Department of Communication, Arts and Media, IULM University, Milan, Italy; 2 Centre Leo Apostel (CLEA), Vrije Universitëit Brussel, Brussels, Belgium; 3 Department of Computer Science and Engineering (Campus of Cesena), Università di Bologna, Cesena, Italy; 4 Department of Biological and Environmental Sciences and Technologies (DiSTeBA), University of Salento, Lecce, Italy

**Keywords:** (M, R)-system, autopoiesis, Church thesis, fabrication, self-reference, turing machine, von Neumann self-reproducing automata

## Abstract

Organizational closure occupies a central position in contemporary theories of autonomy, anticipation, agency, sense making, and the continuity between life and mind. Its investigation has given rise to a diverse landscape of related explanations, including closure to information, cyclic closure, operational closure, semantic closure, closure-to-efficient causation, metabolic closure, and closure of constraints. Although each of these explanatory approaches captures important aspects of organizational closure, their proliferation has often generated conceptual ambiguity. As a result, organizational closure is frequently conflated with feedback loops, recursive algorithms, self-organization, or biochemical cycles. This paper develops a genealogical conceptual analysis of the research programs from which these various accounts emerged, tracing their origins across mathematical logic, first- and second-order cybernetics, general systems theory, thermodynamics, metabolic theory, relational biology, and the biology of cognition. In doing so, it provides a principled framework for assessing the commitments involved whenever a notion of closure is invoked, while clarifying the origins of these explanations and the relations among them. Particular attention is devoted to the roles of self-reference, circular causality, replication maps, semantics, free energy, automata, operations, and constraints in shaping the current understanding of organizational closure. On this basis, the study advances the interpretative thesis that organizational closure is most coherently understood as a circular organization of efficient causes through which living beings continuously produce and maintain the conditions of their own existence. From this perspective, organizational closure appears irreducible to purely algorithmic descriptions inherited from the Church–Turing thesis—not because living beings cannot be simulated in specific respects, but because the causal production of the conditions of their own realization cannot be exhaustively characterized as a state-determined syntactic procedure. This interpretation helps clarify the distinctions between organizational closure and the notions with which it is often conflated, while providing a framework for assessing current debates concerning artificial intelligence, large language models (LLMs), semantic meaning, and sense making in the face of ambiguity and logical paradox.

## 1 Introduction

After Wöhler’s synthesis of urea debunked vitalism, Bernard’s notion of the *milieu intérieur* preserved the boundary between the living and the nonliving. As he wrote, “all the vital processes, varied as they are, have only one object, to preserve the constancy of the internal milieu [which] is the condition for the free and independent life” (Bernard, 1850, p. 84). The constancy of the *milieu intérieur* was later reformulated as homeostasis by Cannon (1929), contributing to the rise of cybernetics and to the algorithmic age of “machine cognition.”

Machine cognition—framed as algorithmic computation—displays increasingly sophisticated behavior, potentially creating the impression that computability exhausts scientific legitimacy: what is computable appears both physical and scientifically sound. Thus far, it remains unclear whether computability alone can account for the condition of living cognition. Even if machine cognition could independently generate scientific knowledge, this would not ensure that it could overcome the epistemological limitations associated with its living counterpart.

Therefore, the constancy of the *milieu intérieur* may instead be reformulated in terms of the constancy of what characterizes living cognition: self-production through organizational closure (Maturana, 1980). However, unlike self-production (a system that fabricates itself), organizational closure, as the causal regime underlying self-production, has remained theoretically elusive. In particular, it appears irreducible to current physical mechanisms whose mathematical formulation is largely algorithmic. Moreover, this theoretical difficulty is compounded by its frequent conflation with feedback loops, self-organization, recursive algorithms, dissipative structures, and autocatalytic or biochemical cycles. Over time, this has led to a proliferation of explanations and the proposal of related notions, including closure to information (Ashby, 1952), replication maps or closure-to-efficient causation (Rosen, 1958, 1991b), cyclic closure (Piaget, 1967), operational closure (Maturana and Varela, 1973), semantic closure (Pattee, 1982), catalytic closure (Kauffman, 1986), metabolic closure (Letelier et al., 2006) and closure of constraints (Montévil and Mossio, 2015).

Although each is significant and valuable for explaining certain aspects of the causal and temporal realization of organizational closure, their proliferation may generate confusion rather than clarification. This issue is particularly pressing given the widespread application of machine cognition in the form of artificial intelligence, machine learning, and large language models (LLMs) across disciplines—from biology and law to astronomy, economics, cognitive science, and climate science.

In this context, a unified explanation of organizational closure is needed. However, it does not lie in selecting a single “best” definition or proposing a final one, but in framing the problem in genealogical terms. Such an approach should not be understood merely as a historical reconstruction of facts, but as a form of conceptual analysis and mapping of origins aimed at identifying how distinct research programs progressively converged on the explanatory problem of organizational closure. This clarification is necessary to avoid conflating computational models of life and cognition with the organizational conditions that make life and mind possible.

This study advances two distinct but connected claims. First, it provides a genealogical conceptual analysis of how different explanations of organizational closure emerged from various research programs, spanning mathematical logic, first- and second-order cybernetics, general systems theory, thermodynamics, metabolic theory, relational biology, and the biology of cognition. Second, it develops the interpretative thesis that organizational closure, as a causal regime, can be understood as a circular organization of efficient causes underlying biological self-production. This distinction is particularly relevant today, as increasingly sophisticated artificial intelligence (AI) systems raise the question of whether adaptive behavior and programed autonomy are sufficient for organizational closure, or whether closure requires additional organizational conditions that current artificial systems do not instantiate.

Section 2 examines the role of self-reference, infinite regress, and logical paradoxes in the foundational crisis of mathematics as formal precursors of organizational closure. Sections 3 and 4 analyze how the search for a physics of mind and life led to causal explanations of organizational closure through different scientific disciplines. Sections 5 and 6 discuss autopoiesis and (M, R)-systems as approaches toward a causal account of organizational closure. Finally, Section 7 synthesizes these genealogies into a diversified landscape of closure explanations and clarifies their implications for current debates on life, cognition, and artificial intelligence.

## 2 The mathematical logic genesis of organizational closure: self-reference, infinite regress, and logical paradox

Two notions—infinite regress and self-reference—proved decisive in the foundational crisis of mathematics. They are relevant here not because they provide a theory of organizational closure, but because they anticipate several conceptual difficulties that later reappear in attempts to understand biological organization, autonomy, and self-production.

Infinite regress arises when explanations demand further explanations ad infinitum, exposing the impossibility of grounding mathematics on entirely self-evident principles. Self-reference occurs when a statement, proposition, or set refers to itself, often giving rise to a logical paradox. A paradigmatic example is the liar paradox, formulated as a self-referential statement.^1^

Logical paradoxes suggest that meaning—particularly concerning truth and falsity—can generate contradictions. Sentences may be grammatically and syntactically correct yet resist the consistent assignment of a truth value. Ultimately, the problems of logical paradox and infinite regress point to deeper issues of sense making. These tensions coalesced in the early twentieth-century foundational crisis of mathematics, giving rise to competing reconstruction programs: logicism (Frege and Russell), formalism (Hilbert), and intuitionism (Brouwer) (Kleene, 1967).

Logicism culminated in *Principia Mathematica* (Whitehead and Russell, 1925), which aimed to demonstrate that “all pure mathematics follows from purely logical premises” (p.27). Hilbert’s Program (Hilbert and Ackermann, 1928) radicalized this project by proposing purely syntactic mathematics governed by three criteria: (i) consistency (absence of contradictions), (ii) completeness (all truths provable), and (iii) decidability (effective procedures exist for every proposition). Both logicism and formalism aimed to eliminate semantics and the ambiguities of self-reference by grounding mathematics entirely in syntax and symbol manipulation.

Gödel’s incompleteness theorems (Gödel, 1931) overturned this aspiration. By encoding meta-mathematical statements arithmetically, Gödel demonstrated that any sufficiently expressive and consistent formal system necessarily contains true statements that cannot be syntactically derived within it. Syntax, therefore, cannot exhaust meaning or eliminate self-reference. Attempts to exclude self-reference instead revealed its structural necessity, rendering both *Principia Mathematica* and Hilbert’s program untenable.

The notion of “effective procedures” was subsequently formalized by Church (1936) and Turing (1936). Church defined them in terms of *λ*-definability and recursive functions, while Turing introduced the abstract mathematical machine that now bears his name. Effective procedures thus became synonymous with algorithmic, rule-based symbol manipulation. Within the interpretation adopted here of the Church–Turing thesis, effective procedures characterize physical phenomena insofar as they are non-self-referential and formally specifiable in the sense envisioned by Hilbert. In this way, the Church–Turing thesis can be seen as extending the aims of *Principia Mathematica* and Hilbert’s program, but jumping from formal systems to natural systems.

Insofar as mathematical languages shape our understanding of scientific knowledge (Gisin, 2020) and therefore sets limits on what can be formally known (Rosen, 1991c), the syntactic conception of mathematics profoundly influenced twentieth-century science. It is therefore unsurprising that modeling natural systems through this specific conception has proved extraordinarily effective (Wigner, 1960). However, this effectiveness—underlying what is now termed “machine cognition”—has been most successful in relation to non-living phenomena. It operates either by excluding self-reference from the formal model of natural systems or by relocating meaning to the observers’ meta-level, thereby making the observer epistemologically external to the observed system.

This tension re-emerges in attempts to understand consciousness, quantum measurement, the origin of life, and even protein folding—domains in which observer, meaning, and biological organization cannot be eliminated. Regardless of computational power, such syntactic systems inherit an observer-independent framework and its associated epistemic constraints. Recent results in machine learning that confront problems of self-reference reveal similar limitations of purely syntactic systems, underscoring the continued relevance of these foundational issues for contemporary AI (Ben-David et al., 2019; Castelvecchi, 2019; Reyzin, 2019).

The foundational crisis of mathematics thus reveals that attempts to eliminate circularity and the observer through syntax alone generate intrinsic limits that reintroduce semantic and organizational considerations. Some of these conceptual tensions reappear, in transformed form, in cybernetics and theoretical biology, where circularity ceases to be merely a formal property of logical systems and becomes a problem of causal organization. The transition from one domain to the other is not deductive. Rather, similar conceptual tensions concerning self-reference, circularity, meaning, and organization emerge in different scientific contexts.

Throughout the remainder of this study, the term “non-computable” does not refer simply to practical computational limitations, computational complexity, or the impossibility of simulation. Rather, it refers to the interpretative claim that organizational closure cannot be exhaustively characterized as a state-determined syntactic procedure in the sense inherited from the Church–Turing thesis. The issue is therefore not whether aspects of living organization can be computationally modeled, but whether the circular production of the conditions of a system’s own realization can be fully captured by algorithmic description.

## 3 The physics of mind: cybernetics, final cause and circular causality

Despite the limits of syntactic formalization revealed by Gödel, and the characterization of effective procedures as algorithms in the Church–Turing thesis, self-reference reappeared in a new form: no longer as an ambiguous and undecidable logical paradox, but as a form of causal circularity capable of characterizing the behavior of biological and artificial systems.

This shift began with two seminal articles published in the same year: *A Logical Calculus of the Ideas Immanent in Nervous Activity* (McCulloch and Pitts, 1943) and *Behavior, Purpose and Teleology* (Rosenblueth et al., 1943). McCulloch later recalled his own interest in *Principia Mathematica* and Pitts’s concern with “problems of circularity, how to handle regenerative nervous activity in closed loops” (McCulloch, 1974, p. 68).

The *Logical Calculus*, developed from Pitts’s study under Rashevsky’s supervision, formalized neural activity as a Boolean network—a discrete analog of Rashevsky’s continuous two-factor model (Rashevsky, 1933, 1939) and structurally comparable to the Turing machine. The three theorems it presented demonstrated that “a net made of threshold devices, formal neurons, can compute those and only those numbers that a Turing machine can compute with a finite tape” (McCulloch, 1974, p. 97). Neurons were thus modeled as binary automata that execute logical operations (and, or, not), linking what was taken to be neural function to finite-automata theory (Kleene, 1956).

**Figure 1:**
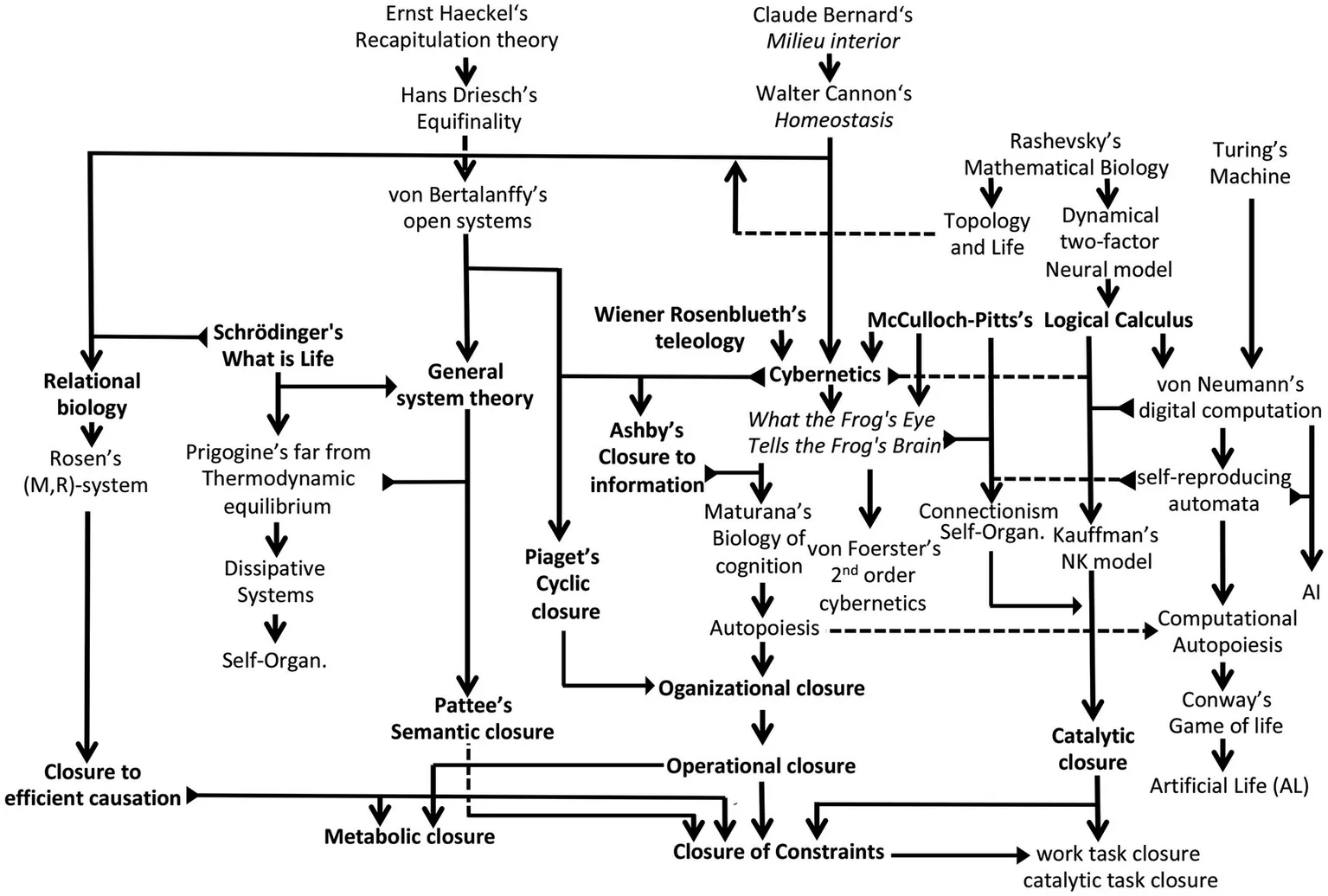
The genealogies of organizational closure. The genealogical scheme represented by arrows follows a timeline (from top to bottom). For example, the studies of von Bertalanffy, Rashevsky, and Turing date from the 1930s, while Ashby’s and Rosen’s closures date from the 1950s and late 1980s, respectively. The intersection of dotted and solid arrows means there is no relationship between them.

This formalization opened the way to the Macy Conferences on cybernetics (1946–1952) and to three major research trajectories: (i) automata-theoretic systems, extended by von Neumann toward digital computation and self-reproducing automata (Von Neumann, 1945; Von Neumann, 1966); (ii) biologically grounded analyses of perception and neural organization, exemplified by *What the Frog’s Eye Tells the Frog’s Brain* (Lettvin et al., 1959); and (iii) Boolean models of genetic regulatory networks, later developed into Kauffman’s NK framework (Kauffman, 1971). Each contributed, in distinct ways, to the growing plurality of explanations of organizational closure (Figure 1).

In parallel, Behavior, Purpose, and Teleology sought to unify engineering and physiology. Wiener, motivated by anti-aircraft targeting through self-correction, and Rosenblueth, studying the neurophysiology of “intentional tremor,” converged on a framework in which systems—biological or artificial—were described through closed loops linking sensors, effectors, and comparators. Drawing on Cannon (1929), this framework reintroduced teleology into science, redefining it as goal-directed behavior grounded in circular causality.

In this reconceptualization, the long-exiled Aristotelian final cause, considered metaphysical, was reintroduced into scientific discourse as teleological behavior. Teleology was immediately redefined in terms of the closed loops or circular causality, in which sensors were redefined as inputs and effectors as outputs of signal processing. With the incorporation of Shannon’s information theory (Shannon, 1948), signals became purely syntactic, stripped of semantic content.

The introduction of teleology together with neural networks marked a shift from purely energetic explanations to those involving information processing and causal organization of inputs and outputs. This move carried forward the residues of the foundational crisis in mathematics into the organizational domain of natural systems. The seeds of cybernetics were sown through the Macy Conferences (1946–1952). These conferences were explicitly devoted to exploring “the physical bases of mind” (Heims, 1991). An early articulation of such bases comes from Wiener, who coined the term cybernetics: “The central nervous system no longer appears as a self-contained organ, receiving inputs from the senses and discharging into the muscles. On the contrary, some of its most characteristic activities are explicable only as circular processes, emerging from the nervous system into the muscles, and re-entering the nervous system through the sense organs” (Wiener, 1948, p. 24).

However, in the cybernetic assimilation, a teleological circularity of mind and behavior was recast as a feedback mechanism—a concept transplanted from Maxwell’s analysis of the centrifugal governor. In this transfer, feedback was transposed from the realm of machines to the organization of the nervous system and mind. This assimilation was taken for granted not only by Wiener but also by the majority of the Macy Conferences’ participants. Circular causality was thus equated with feedback loops, enabling the modeling of self-regulation and homeostasis through error correction. This culminated in Ashby’s homeostat state-determined system (Ashby, 1952) and the Conant–Ashby theorem, establishing that every good regulator must embody a model of the system it regulates (Conant and Ashby, 1970). First-order cybernetics thus operationalized the mind as feedback-based self-regulation:

$$\begin{array}{l} \text{Physics}\to \text{Circular causality}=\text{Feedback}\\ \to \text{Self}-\text{regulation}\;(\text{teleology})\to \text{Mind} \end{array}$$

^2^

This equation became the hallmark of first-order cybernetics and shaped early AI and the mechanization of the mind (Dupuy, 2009).^3^ Still tensions soon emerged. Von Neumann’s automata theory (Von Neumann and Burks, 1966),^4^ connectionist models, such as the perceptron (Rosenblatt, 1958) and experimental results on the neuroscience of perception (Lettvin et al., 1959), all challenged purely sequential symbolic computation, emphasizing distributed, networked, and self-organized emergent processes.^5^ Nevertheless, these approaches, except the experimental results, remained within the computational paradigm and consistent with the Church–Turing thesis.

A critical tension became explicit in the introduction of the last volume of Macy conferences, where von Foerster described circular causal processes as “a state reproducing itself, like an organism” (von Foerster 1952, p. 13), suggesting a form of closure not reducible to feedback. Thus, while the concept of organizational closure inherits the notion of circular causality from cybernetics, it also exposes its limitations—namely, the mechanization of causality and the absence of semantics. This missing dimension re-emerges when biological organization becomes central again to explaining life and its physical basis.

## 4 The physics of life: general system theory, open systems and thermodynamics

With the rise of cybernetics, the need to understand the physical basis of life grew. However, whereas cyberneticists sought the purpose of mind, theoretical biologists and physicists increasingly grounded the purpose of life in maintaining bounded internal conditions as an end in itself.

Biologists and physicists approached this problem from different directions. For physicists, it was formulated in terms of the emergence and maintenance of order and low entropy—a lineage extending from Boltzmann to Erwin Schrödinger, Nicholas Rashevsky, and Ilya Prigogine (Figure 1). Even within physics, however, two distinct approaches can be identified.

One influential formulation appears in Schrödinger’s *What is Life?*, where he asked: “how can the events in space and time which take place within the spatial boundary of a living organism be accounted for by physics and chemistry?” (Schrödinger, 1945, p. 47). Schrödinger’s answer centered on negative entropy and the code-script. More recent developments have reformulated negative entropy in terms of anti-entropy, understood not simply as a reduction in thermodynamic entropy but as a component of Gibbs free energy associated with the emergence of multilevel, integrated, and regulated biological organization (Bailly and Longo, 2009). Earlier attempts in this direction can be found in the study of von Foerster and Atlan, both of whom sought to characterize biological organization beyond classical thermodynamic descriptions, including those based on Shannon’s information theory (von Foerster, 1960; Atlan, 1972a,b).

However, both Rosen and Maturana remained critical of Shannon’s theory as a basis for understanding living organization (Rosen, 1977; Maturana, 1983), and Rosen was equally skeptical of explanations grounded solely in Gibbs free energy (Rosen, 1964). For Rosen, the crucial issue in Schrödinger’s study was not negative entropy or information per se, but the code-script beyond a purely codon–genomic interpretation (Rosen, 1999; Rubin, 2017). In simplified form, Schrödinger’s proposal may be summarized as:

Code−script→Order from order→Negative entropy→Life

^6^

More recently, the reformulation of negative entropy through the Free Energy Principle (FEP) (Friston, 2013; Ramstead et al., 2018), also known as active inference, appears more naturally related to the relational domain of living systems—that is, cognition—than to their organizational constitution (Rubin, 2023). More study is needed, however, to clarify the relationship between the physics presently available to us and the “new physics” that Schrödinger anticipated as necessary for understanding life. In this respect, the analyses developed by Bailly and Longo (2011) on the singularity of life offers an important contemporary contribution to understanding the relationship between physics and biology in connection with the foundational problems of mathematics.

Simultaneously with Schrödinger’s study, Rashevsky developed reaction–diffusion systems as models for the emergence of order (Rashevsky, 1940), while Turing later reformulated the problem in terms of chemical morphogenesis (Turing, 1952) and Prigogine in terms of irreversible thermodynamics and dissipative structures (Prigogine and Nicolis, 1967). At first glance, this trajectory may be condensed as:

$$\begin{array}{l} \text{Reaction}–\text{diffusion}=\mathrm{far}-\text{from}-\text{equilibrium}\\ \text{thermodynamics}\to \text{order}\to \text{Life} \end{array}$$

However, order and organization should not be regarded as equivalent concepts. In physics, order is typically understood in terms of entropy reduction or symmetry breaking in open or closed systems. In biology, by contrast, organization refers to “the relations between components which define a system as a unity,” such that it “determines their invariant class identity” (Maturana, 1975, p. 82). Rashevsky made this point forcefully: to understand living systems, one must “keep the organization and throw away matter” (Rashevsky, 1954, p. 97). The genealogy of organizational closure therefore begins with the recognition that biological organization is irreducible to physical order. As Rosen later remarked, “we need a physics of organized matter” (Rosen, 1991b, p. 196).

Order≠Organization→Life

Within biology, organization was historically tied to the problem of form, and finality was framed in terms of the origin and persistence of form. This perspective was emphasized in the study of Ernst Haeckel and Hans Driesch,^7^ and contributed to the establishment of theoretical and mathematical biology through the study of D’Arcy Thompson, Ludwig von Bertalanffy, and Conrad Waddington.

Driesch’s sea urchin experiments in 1891 revealed the phenomenon of equifinality: separated blastomeres developed into complete organisms. In the absence of a mechanical explanation, Driesch invoked Aristotle’s concept of *entelechy*.^8^ As early as 1928, von Bertalanffy identified equifinality with the stability of point attractors in dynamical systems (von Bertalanffy, 1950).^9^ Equifinal phenomena could therefore be explained through steady states (*Fließgleichgewicht*) and attractors in open systems, recognizing that the equilibria of closed systems are not attractors in the dynamical sense. This is because closed systems lack the sustained dissipation or external driving required to pull trajectories toward a particular state over time (von Bertalanffy, 1972). Such a perspective reconciled the second law of thermodynamics with the biological problems of form and organization.

$$\begin{array}{l} \text{Open systems}\to \text{Steady states}\to \text{Point attractors}\\ (\text{equifinality})=\text{Form and organization} \end{array}$$

As Rosen (1999) later remarked, Driesch’s mistake was to conflate “physics” with the physics of closed systems.^10^ Three central lessons follow.

- Open systems are more general than closed systems, both physically and mathematically.

Thermodynamic equilibrium≠Dynamical attractor

- Nevertheless, they remain under-theorized, as “people persist in thinking of closed systems as fundamental, and of open ones simply as closed ones canonically disturbed” (Rosen, 1985c, p. 71).

Open systems≠Thermodynamically perturbed closed systems

- Mathematically, open systems generally lack the symmetries and conservation laws characteristic of closed systems. However, Rashevsky (1940) demonstrated that they can spontaneously generate morphogenetic gradients, anticipating both Turing’s theory of chemical morphogenesis (Turing, 1952) and Prigogine’s symmetry-breaking dissipative structures (Prigogine and Nicolis, 1967). A vast literature on pattern formation and physicochemical self-organization has subsequently emerged from these ideas.

By the 1950s, the theory of open systems provided the foundation for General System Theory (GST), which became central to systems analysis (von Bertalanffy, 1968).^11^ Modern systems biology, systems chemistry, and systems ecology continue to draw upon the GST tradition. At the same time, far-from-equilibrium thermodynamics and entropy theory opened new avenues for theoretical biology and the search for universal explanations of living organization.^12^

The crucial point is that the conception of biological systems as dynamically open systems—rather than merely thermodynamically open ones—provided the antagonistic yet complementary conceptual background from which the notion of biological systems as organizationally closed systems would later emerge.

## 5 The Biological Computer Laboratory (BCL): from biology of cognition and second-order cybernetics to autopoietic operational closure

The Biological Computer Laboratory (BCL), founded by Heinz von Foerster in 1958,^13^ offered an intellectual background from which the notion of biological systems as organizationally closed systems would emerge. This turn was first elaborated by W. Ross Ashby as “systems that are open to energy but closed to information and control – systems that are ‘information tight’” (Ashby, 1958, p. 154). Although Ashby did not officially join the BCL until 1961, his study was decisive for the development of organizational closure. The argument that the environment does not specify internal organization, and that what appears as an “input” is internally determined (von Foerster, 1960), directly follows this closure-to-information idea.

In the context of the BCL, circular causality returned to the ancient roots from which the lineage of organizational closure draws its sustenance. As von Foerster mentioned: “Our interest, at BCL, was independent of the main notions of cybernetics, which, I must say, seemed to me—at the time it was ‘first order cybernetics’—some people now call it ‘thermostat cybernetics’—rather unfascinating. What fascinated me was circular causality. The notions of feedback, positive, negative, control, ok, interesting, but…” (von Foerster et al., 1985, p. 267).

The circular causality to which von Foerster referred was precisely tied to the foundational crises of mathematics discussed in Section 2—problems that first-order cybernetics, constrained by Russell’s theory of types, had avoided. This avoidance led to the rise of AI as a separate field from cybernetics, reducing the latter to “control theory applied to living organisms” (Franchi et al., 1995, p. i). Accordingly, “cybernetics must be the point where one can overcome Russell’s theory of types by taking a proper approach to the notion of circularity; whether in the form of circular causality or in the form of the argument itself or in the form of paradox and self-reference” (von Foerster and Pakman, 1996). In other words, shifting the focus of circular causality toward self-reference, rather than equating it merely with feedback loops, was a fundamental move from which organizational closure would emerge:

Circular causality=Self−reference≠Feedback

However, this move presented a risk. Ashby’s notion of systems “closed to information” could not offer a causal thesis for self-reference; it remained a mathematical formalism based on Shannon’s syntactic information. This limitation was overcome by the concept of material “cyclic closure,” which the biologist Jean Piaget posited as a necessary complement to von Bertalanffy’s open systems. He argued: “[T]he central ambiguity is that of the ‘open system’, for, if the system exists, then something like a closure intervenes. This cyclic closure and the opening of exchanges are, therefore, not on the same plan […] the cyclical character of the system is particularly necessary since the organization takes the form of adaptation and assimilation” (Piaget, 1967, p. 397).

Because Piaget was influenced by cybernetics and General Systems Theory (GST), this cyclical closure was indispensable for both biological regulation and cognitive organization (Damiano, 2012). Although he was never a member of the BCL, his study closely coincided with that of Maturana and Varela, who were visiting researchers at the BCL.

Similarly to Piaget, Maturana considered von Bertalanffy’s open systems necessary but insufficient, seeking the key to living beings in their condition as autonomous systems.^14^ Based on experimental visual results that undermined the plausibility of information-processing and representational accounts of perception, he concluded that perception arises from action–perception (sensorimotor) loops (Maturana, 1969).^15^ From this, Maturana inferred that neuronal activity must be organized as a closed system, analogous to the organism’s metabolic organization, thereby linking circular causality to autonomy as an internally determined process of dynamic material production.

Living beings were thus understood as “self-referred” systems, and the nervous system as capable of generating its own conditions of reference. This framework was soon after termed the “Biology of Cognition,” suggesting that “living systems are cognitive systems and living as a process is a process of cognition. This statement is valid for all organisms, with or without a nervous system” (Maturana, 1970, p. 13). In a manner broadly consistent with the *Umwelt* tradition, the Biology of Cognition maintains that perception—whether in organisms with or without a nervous system—is not the computational processing of objective information through syntactic rules applied to an observer-independent world. Rather, cognition emerges through embodied perception–action loops grounded in organizational closure, in which the central problem is selecting the next action (Valdés-Zorrilla et al., 2023, 2024).

However, before reaching these conclusions, the notion of self-reference proved unsatisfactory to Maturana because it seemed to imply a pre-existing totality.^16^ This difficulty led to the conception of autopoiesis in collaboration with Varela,^17^ who later recalled: “The question under examination then was: if we leave the organization of the nervous system to the side for the moment and focus on the autonomy of life in its cellular form, what can we say? This reflection on the circular nature of metabolism in living beings and its relation to cognitive operations, although barely filling a short page in the definitive version of ‘Biology of Cognition’, would be a focal point from which the development of the idea of autopoiesis would be drawn” (Varela, 1996b, p. 412).

The autopoietic characterization of biological organization moved circular causality beyond feedback, open systems, formal logical paradoxes, and pure self-reference into a closed organization of material (metabolic) production (Maturana and Varela, 1973). This self-production ultimately equates life with mind, putting autonomy at the center of biological phenomenology (Thompson, 2010):

$$\begin{array}{l} \text{Circular causality}=\text{Internally determined closed}\\ \text{organization of material production}\to \text{Mind} \end{array}$$

$$\begin{array}{l} \text{Circular causality}=\text{Internally determined closed}\\ \text{organization of material production}\to \text{Life} \end{array}$$

Life=Mind

In this move, Warren McCulloch’s experimental epistemology of mind—an approach aimed at preserving the role of the observer—was key (McCulloch, 1965; Van de Vijver, 1992). Autopoiesis emerged from this lineage, via the Biology of Cognition, as a direct response to the representationalist challenges posed by first-order cybernetics, Shannon-based information processing, and the broader computationalist paradigm of symbolic AI, none of which adequately safeguarded the autonomy of the cognitive being. These traditional explanations systematically excluded the lived experience (the *living*) of the observer, or alternatively reduced it to a subjectless feedback loop mechanism, an automaton, or a Turing machine.

One attempt to overcome this limitation was the establishment of second-order cybernetics (Froese, 2010), championed by von Foerster (1976). This framework presents the observer mathematically through the emergence of self-referential processes, leading to the coordination of coordinations observed as stable *Eigen-behaviors*. Nevertheless, this was not entirely sufficient; the older problems of the foundational crises (see Section 2) were merely displaced rather than solved. Symbolic AI became a renewed attempt to assert that all natural systems, including living systems and their phenomenology, are exclusively syntactic and observerless.

In Autopoiesis these problems involve the very core of biological organization. The remarkable solution it puts forward is to operationally identify the observer as an entity whose being is its own doing via self-production (Maturana and Varela, 1987; Froese, 2011). This solution resonates with Gödel’s incompleteness theorems, which evaluate arithmetic systems through an observer meta-system—a perspective that was otherwise obliterated by the Church–Turing thesis (Penrose, 1990; Rosen, 1991b; Rubin, 2023).

Paradoxically, the first formalization of autopoiesis, in the sense of Hilbert’s program, relied on computable models based on von Neumann’s self-reproducing automata, where essential catalysts were supplied from outside the system (Varela et al., 1974). By this time, the inadequacy of the self-reproducing automaton as a model of living systems had already been established on formal grounds by Rosen (1959),^18^ a conclusion subsequently echoed by Ashby (1962a).^19^ This insight influenced Varela’s future modeling of autopoiesis via Lars Löfgren (1968),^20^ who argued that while Rosen’s proof is correct, self-reproduction could remain consistent if specified from an independent axiomatic system external to the automaton itself.

This external dependency for the consistency of a system’s self-reproduction was likely a source of dissatisfaction for Varela, who later pursued alternative formal systems for autopoiesis based on George Spencer-Brown’s calculus of indications (Spencer-Brown, 1969). Varela demonstrated that autopoietic self-reference arises autonomously through self-indication (Varela, 1975). While this result strengthened the solution to the observer problem in formal terms, it lacked the causal (physical) grounding required to be realized as a natural system capable of overcoming the limitations of the autopoietic automata—yet it remains evocative of a non-computable form of organizational closure (Rubin, 2023).

It is on this basis that the notion of organizational closure began to shape the “languaging” among BCL members and research visitors.^21^ Varela first published the closure thesis stating that “every system-whole is organizationally closed” and “every autonomous system is organizationally closed” in his *Arithmetic of Closure* (Varela and Goguen, 1978) and *On Being Autonomous* (Varela, 1978), respectively. These statements were highly significant because they indicated that autonomy is constitutive of organizational closure, and thus of the internal causation of self-production (Thompson, 2010).

Varela’s synthesis of organizational closure—drawing largely on Ashby’s closure to information, Piaget’s cyclic closure, Maturana’s closed causal organization, von Foerster’s second-order cybernetics, and Bateson’s (1979)—fully crystallized in *Principles of Biological Autonomy* (Varela, 1979). This synthesis carries the semantic legacy of the foundational crisis in mathematics (namely, self-reference) while integrating key insights from general systems theory, cybernetics, relational biology, and the biology of cognition.^22^ Organizational closure was subsequently specified as *operational closure* by Maturana and Varela (1987).^23^

After the dissolution of the BCL, the concept of organizational closure disseminated across several institutions and alternative intellectual venues, notably the Lindisfarne Association (1972–2012) (Thompson, 1987, 1991).^24^ Within Lindisfarne, Varela encountered early drafts of Bateson’s *Mind and Nature* Bateson (1979), while Lynn Margulis recognized the relevance of autopoiesis for Gaia theory and biology more broadly (Margulis and Sagan, 1986; Margulis, 1997; Rubin et al., 2020, 2021). These engagements also fostered a sustained dialogue with Zen Buddhism, contributing to the founding of the Mind and Life Institute (Hayward and Varela, 1992), as well as the emergence of neurophenomenology (Varela, 1996a) and contemplative neuroscience (Barinaga, 2003).

## 6 The Center of Theoretical Biology (CTB): from biophysics and relational biology to closure-to-efficient causation

Nicolas Rashevsky articulated a bold vision to “bring mathematical biology to the same level as mathematical physics” (Rashevsky, 1938, p. 168) well before the advent of the Macy Conferences, General Systems Theory (GST), and Erwin Schrödinger’s foundational study. This program led to the founding of the *Bulletin of Mathematical Biophysics* (hereafter *the Bulletin*) in 1940, a journal that would go on to attract prominent figures such as Walter Pitts, Warren McCulloch, Marvin Minsky, Robert Rosen, or more recently Fontana and Buss (1994). As a teenager, Pitts joined Rashevsky’s group and refined his continuous neural two-factor network model—a collaboration that served as a direct precursor to the seminal McCulloch–Pitts “Logical Calculus” study (McCulloch and Pitts, 1943), published in Rashevsky’s *Bulletin*. Rashevsky’s study during the 1930s and 1940s was remarkably prescient, extending beyond neural networks to areas such as chemical diffusion–reaction systems (Rashevsky, 1940), a core concept of self-organization that preceded Alan Turing’s study on morphogenesis and Ilya Prigogine’s theory of dissipative structures by decades.^25^

Following the publication of Schrödinger’s *What is Life?* (1945), Rashevsky turned his attention to the same central question of biology. He proposed a radical shift in perspective: to understand the organism, one must discard the material and retain the underlying organization (Rashevsky, 1954). This approach, which he termed Relational Biology, was based on a “bio-topological mapping,” which posits that “all organisms can be mapped on each other in such a manner that certain basic relations are preserved in this mapping” (Rashevsky, 1954, p. 323), independent of their material constitution. The ultimate goal was to identify a universal “organization chart” or pattern of metabolic functions common to all life, whereby complex processes in higher organisms could be understood as mappings of simpler processes in lower organisms (Rashevsky, 1956; Bich and Damiano, 2008):

$$\begin{array}{l} \text{Organization chart}\to \text{Universal topological}\;\\ \text{mapping of metabolism}(e.g.,\text{Ingestion}\to \text{Metabolism}\to \text{Excretion}) \end{array}$$

This relational approach, distinct as it was from cybernetics, GST, or molecular biology, was frequently misunderstood. Norbert Wiener, for instance, criticized Rashevsky’s school for being “too dominated by problems of energy and potential and the methods of classical physics” (Wiener, 1948, p. 13). His former student, Robert Rosen, later defended Rashevsky, characterizing Wiener’s remark as “a total, and almost certainly a willful, misconstruction” (Rosen, 1991b, p. 112), especially given that Pitts’s Boolean networks were fundamentally a discrete version of Rashevsky’s own continuous two-factor network models.

Rosen found in Rashevsky’s Relational Biology the methodological pathway to search for the common organization of living systems. Utilizing the mathematically powerful language of Category Theory—which permits the analysis of formal logic and comparisons between formal systems—Rosen developed the metabolic-repair (M, R)-system in 1958 as a definitive response to the problem of infinite regress in biological organization (Rosen, 1958). As he later noted: “My adaptation of Category Theory to the (M, R)-system… for the notion of modelling in general…[was]… an invocation of the entailment patterns from which the theorems arise, or sometimes do not arise.” (Rosen, 2006, p. xv).

The (M, R)-system is a model that characterizes the organization of a living unity in terms of causality. It identifies a metabolic function (M) that processes environmental materials or signals, and a repair function (R) that produces the components of the metabolic function M, while M subsequently produces R via a replication map (Rosen, 1972b).

From the perspective of the (M, R)-system, the problem of infinite regress arises as follows: if a catalyst F produces B from an environmental milieu A, there must be another catalyst F_1_ that produces F from B, and then another catalyst F_2_ that produces F_1_ from F, and so on, leading to an incipient infinite regress. Crucially, the (M, R)-system achieves a resolution of this infinite regress through an internally produced (though not physically isolable) replication map that closes the system upon itself. This closure makes the distinction: “[B]etween metabolic, repair and replication activity… not an absolute distinction… that is, a given (M, R)-system could in principle be extended in such a way that the original metabolic components became repair components and the original repair component became replication components” (Rosen 1978c, pg. xv), thereby entailing the system’s self-fabrication. Rosen identified the catalysts F to F_n + 1_ with the active sites of enzymes (Rosen, 1972a).^26^

$$\begin{array}{l} \text{Active sites}\;(\text{catalyst}\;F\;\text{to}\;F_{n+1})\to \text{Replication}\;\mathrm{map}\to \\ \text{Entailment of self}-\text{fabrication} \end{array}$$

To characterize biological organization through causality, Rosen adopted the Aristotelian categories of causality—material, formal, efficient, and final—which were not a philosophical caprice but a deliberate mathematical choice (Rosen, 1985d).^27^ This enabled him to identify the replication map with efficient causation, leading to his central theorem: “a material system is an organism if, and only if, it is closed to efficient causation” (Rosen, 1991b, p. 244).

Replicationmap⇔Closure−to−efficient causation

This represents the most critical conceptual shift away from pure self-reference, circular causality, and autopoietic operational closure. Here, circular causality is understood not merely as the circular organization of efficient causes, but as the circular, structural interchangeability of material, formal, final, and efficient causes within the same organizational framework. That is, the structural realization of biological organization encompasses all four causal categories, but its components are not rigidly fixed to any single one of them.

$$\begin{array}{l} \text{Circular causality of efficient causes}=\text{Interchangeability}\\ \text{of material},\text{formal},\text{final},\text{and efficient causes within the}\;\\ \text{same structure} \end{array}$$

This circular and constructive dynamic cannot occur in non-living natural systems. In other words, the self-fabrication of a living system means that its efficient causes must be produced within the system and, at the same time, possess the structural plasticity to be transformed into the other three causes.

This conclusion allowed Rosen to demonstrate that von Neumann’s self-reproducing automaton is a logically inconsistent model of living systems (Rosen, 1959) because it conflates efficient causes with material causes, and a universal computer with a universal constructor (Rosen, 1985b).^28^ Furthermore, it enabled the identification of the strictly syntactic nature of state-determined systems (mechanisms) and highlighted their inability to incorporate final causes—and, consequently, a semantic component that had been excised from science since the rise of Newtonian mechanics (Rosen, 1985d).

Accordingly, a material system is considered alive if it embodies the (M, R)-system model, which also entails, on formal grounds, final causation expressed as anticipation (Rosen, 1985a, 2022; Nadin, 2010; Louie, 2012). This insight led him to prove that standard dynamical systems could not formally model biological organization (Rosen, 1971, 1973, 1991a), systems far from thermodynamic equilibrium (Rosen, 1978b), or cybernetic feedback systems, due to their structural dissimilarity with the (M, R)-system. In short, living systems are physical (causal) systems that are not mechanisms (Rosen, 1985d).

$$\begin{array}{l} \text{Living systems}=(M,R)-\text{systems}\;(\text{Complex}/\text{Non}-\text{Computable})\\ \neq \text{Mechanisms}\;(\text{Self}-\text{reproducing automata},\text{dynamical systems},\\ \text{cybernetic systems},\text{Turing machines}) \end{array}$$

**Figure 2:**
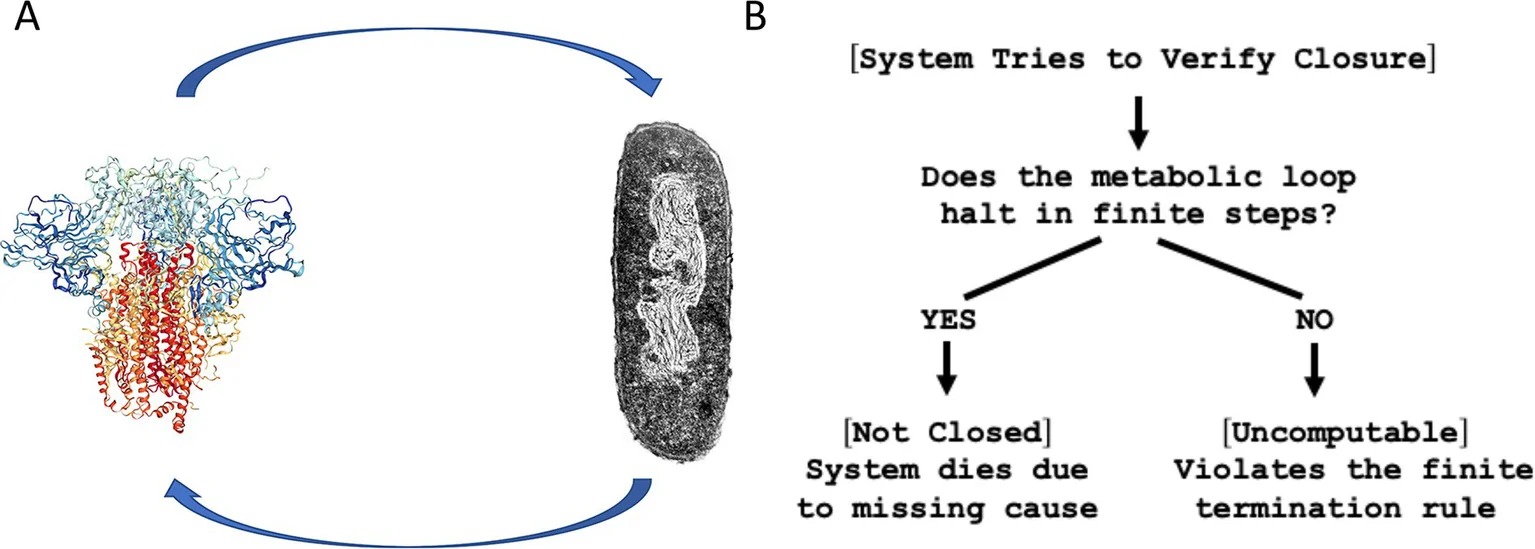
The protein folding problem, organizational closure and impredicativity. **(A)** A paradigmatic example of organizational closure in the causal chain of the protein folding. Cells cannot maintain their organization without folded catalytic sites of enzymes, and enzymes cannot fold their catalytic sites outside the invariant cell organization. **(B)** Organizational closure and the halting problem.

As such, the (M, R)-system directly challenges the Church–Turing thesis and the tractability of natural systems by syntax alone (Rosen, 1962; Louie, 2020). Organisms serve as existential proof of natural systems—living systems—that physically realize self-reference outside the domain of Turing machine models and Newtonian mechanics (Rosen, 1985d, 1988; Louie, 2007; Lane, 2024). Rosen considered the folding of enzymes to be a paradigmatic example of self-production via closure-to-efficient causation (Rosen, 1991b) (Figure 2A). This implies that protein folding involves non-trivial semantics and that the three-dimensional molecular phenotype of a folded protein is essentially non-syntactic (non-algorithmic) in character.

The final protein shape cannot be deduced or predicted solely from the potential physicochemical landscape of the folding configuration, the DNA sequence, or the translated polypeptide sequence. Recent breakthroughs from AlphaFold, which still do not achieve 100% molecular phenotypic identity with laboratory-crystallized proteins, are statistical approximations based on extensive databases of experimentally determined structures. Without these physical crystallization datasets, it would be impossible to obtain such *in silico* approximations. The folding protein is inherently impredicative or “paradoxical” within the syntactic, predicative domain of the computable approach utilized by AlphaFold. In the context of the origin of life, this limitation is tied directly to the “ground zero” problem (Luisi, 2014). In the context of evolution, where modifications to protein structures generate functional novelty (such as the emergence of Rubisco from evolutionary precursors, which led to the production of atmospheric oxygen and the planetary Great Oxidation Event), closure-to-efficient causation intrinsically embodies final causation in the form of anticipation.^29^

Rosen’s oeuvre has generated extensive debate, particularly concerning the implications of impredicativity, closure-to-efficient causation, anticipation, and their relationship to computability. The present study does not seek to adjudicate these controversies or to provide a definitive assessment of Rosen’s formal claims; such questions remain active topics of discussion within theoretical and philosophical biology. Instead, this study reconstructs his contribution as one of the most influential attempts to formulate organizational closure in causal terms and to connect biological organization with the broader problems of computation, meaning, and autonomy. From a genealogical perspective, Rosen’s significance lies not only in the specific conclusions he reached, but also in the rich conceptual landscape that his study helped establish.

Recent attempts to mathematically disprove Rosen’s claim—namely, that organisms cannot be fully represented by simulable models—remain inconclusive. Aloisius H. Louie (2007) has raised substantial objections to several proposed refutations, particularly those advanced by Chu and Ho (2006). In several cases, the appearance of a contradiction stems from replacing Rosen’s carefully formulated definitions with broader or less rigorous alternatives. For instance, Mossio et al. (2009) adopt a notion of computability that does not require a computation to terminate after a finite number of steps. Crucially, if we enforce the requirement that every computation must terminate in a finite number of steps, the Mossio et al. (2009) model of closure-to-efficient causation collapses into uncomputability. Under this condition, the system directly triggers the Halting Problem, thereby proving Rosen’s original conjecture that true metabolic closure cannot be simulated on a standard, terminating Turing machine (Figure 2B).

The Halting Problem states that it is impossible to write a general algorithm to decide whether an arbitrary program will halt or run indefinitely. When applied to finite closure, a fatal paradox emerges:

- The Decidability Paradox: To prove a system has achieved valid closure within a finite timeframe, an external or internal supervisor must verify that all causal loops successfully close without entering an infinite, non-functional hang.
- The Undecidability Mapping: Determining if a self-referential graph of metabolic and repair structures will successfully resolve all dependencies in finite time maps directly to checking if a program halts. As the Halting Problem is undecidable, predicting or guaranteeing finite closure for an arbitrary relational system is computationally impossible.

Shifting to a strictly terminating view of computation alters our understanding of biological simulation in three ways:

- Rosen Is Vindicated: Robert Rosen’s claim that (M, R)-systems are non-computable holds true if and only if computability is defined by halting algorithms.
- Living Systems as Non-Halting Processes: It forces us to categorize biological closure not as a static “result” or “output,” but as a permanently running, non-halting process.
- Simulation Limits: Computer simulations of life on digital hardware cannot be truly autonomous; they are merely “frozen” approximations or finite unrollings of an infinite causal chain.

Moreover, although computability as defined by Mossio et al. (2009) is widely accepted within computer science, a more fundamental issue lies in its formal reconstruction of Rosen’s framework, which relies on a set of equations that does not accurately represent the original scheme. However, these conclusions remain provisional, as further research is required to elucidate how the explanation of organizational closure relates to different notions concerning: (i) the limits of computation, (ii) the limits of formalization, and (iii) the boundaries of a given scientific paradigm (Table 1).

**Table 1:** Related notions of non-computability raised by the genealogy of organizational closure, along with their distinct empirical and mathematical consequences.

Claim	Meaning	Mathematical consequence	Empirical consequence
(i) Not turing-computable	No algorithm can generate the system’s behavior	Exceeds the church–turing framework	Simulation may fail in principle
(ii) Not formally axiomatizable (Hilbertian)	No finite formal system can completely capture the system	Gödelian incompleteness; undecidable truths remain	Complete theoretical description is impossible
(iii) Not adequately modeled by classical computational cognitive science	The system cannot be explained solely as syntactic information processing in a state-determined machine	Computational models may be mathematically valid but explanatorily insufficient	Cognition, meaning, autonomy, or agency remain unexplained despite successful prediction

A system may be Turing-computable yet remain unexplainable by computational cognitive science (iii), and it may be computationally simulable while still resisting complete axiomatization (ii). Rosen’s strongest claims concern positions (i) and (ii) through closure-to-efficient causation, whereas Maturana, Varela, and contemporary enactive approaches primarily challenge position (iii), arguing that life and mind require organizational closure, autonomy, and intrinsic meaning beyond pure syntactic computation.

The central implication of Relational Biology is the potential extension of current physics derived from an understanding of organizational closure, echoing Schrödinger’s ultimate conclusion that understanding life requires a “new physics” (see Section 7). That is to say, computable and algorithmic processing—being purely syntactic—becomes the specific case, while organizational closure represents the general case. Rosen and his student Louie refer to these systemic properties as the definition of complexity itself (Rosen, 1988, Rosen, 1991a,b; Louie, 2007, 2009):

$$\begin{array}{l} \text{Closure}-\text{to}-\text{efficient causation}\\ (\text{Organizational closure})=\text{Complex systems} \end{array}$$

Although we may acknowledge that certain elements of biological phenomenology are formalizable by computational (i.e., mechanistic and syntactic) means, this does not imply that organisms are constrained by the limits of computable (Turing) models. Complexity as a non-algorithmic phenomenon implies a profound revision of foundational assumptions across cognitive science (Miller et al., 2025; Rodriguez-Vergara and Husbands, 2026), enactive evolutionary theory (Maturana and Mpodozis, 1991; Varela et al., 1991; Mpodozis, 2022; Rubin, 2022), cell biology (Hofmeyr, 2007; Vega, 2024), and Earth system functioning (Rubin and Crucifix, 2021, 2022; Rubin et al., 2021), as well as future developments in synthetic biology and artificial intelligence.

## 7 Post BCL and CTB period and future discussion

It remains unclear whether Rosen was aware of autopoiesis and its similarity to (M, R) systems. Varelas’s *On Being Autonomous* (Varela, 1978) was published in the book *Applied General Systems Research* edited by Klir (1978),^30^ which also included contributions by Rosen (1978a), Pattee, and Löfgren. As far as we know, this was the first time that Rosen and Varela’s study intersected with the publication of Klir’s edited volumes, first Rosen’s *Fundamentals of Measurement and Representation of Natural Systems* (Rosen, 1978c) and subsequently Varela’s *Principles of Biological Autonomy* (Varela, 1979). Varela was well acquainted with some of Pattee and Rosen’s study on dynamical systems theory in biology, which appears to be cited in Varela (1979). Pattee was also well acquainted with the work produced by BCL members and visitors, having personal contact with them and Varela. Pattee worked closely with Rosen at CTB for at least a decade (Pattee, 2007). Given the overlap of publications of members of the CTB and the BCL that occurred not only through Klir’s edited volumes, but also through venues such as the *Journal of Social and Biological Structures*,^31^ where publications of Rosen, Pask, Maturana, and Varela became increasingly intertwined, Rosen’s familiarity with autopoiesis, and therefore with the emerging notion of organizational closure, appears highly plausible.

The contributions of Varela (1986), and Rosen (1986), among others, including Beer and von Glasersfeld, in other collections (Trappl, 1986), completed the configuration of a more elaborate explanation of the closed organization of living systems. During this period, Rosen progressively reformulated the replication map in terms of closure-to-efficient causation, while Varela gradually distanced his study from the language of cybernetics. The closure explanation began to establish itself as a powerful tool intended to fill an important gap in the biological and cognitive sciences. Around this period, Pattee introduced the concept of “*semantic closure”* (Pattee, 1982), introducing, after Ashby, constraints as a central explanatory category and describing an organizational loop between symbolic constraints, which he considers to be imprinted in the DNA sequences, and physical dynamics, which correspond to Newtonian state-determined transition mechanisms. This idea sought to address von Neumann’s realization problem for self-reproducing automata by showing how constraints as syntactic rules in DNA sequences could both construct and interpret their own functional context (Pattee, 2008).

$$\begin{array}{l} \text{Semantic closure}=\text{Symbolic constraints}\;\\ (\mathrm{DNA}\;\text{syntactic rules})\Leftrightarrow \text{Physical dynamics} \end{array}$$

Pattee’s semantic closure, however, does not use nor refute Rosen’s theoretical argument against self-reproducing automata (Rosen, 1959). This was followed by the development of the NK model of gene expression inspired by Pitts-McCulloch’s Boolean “logical calculus” network by (Kauffman, 1971).^32^Based on this model and the Hypercycle (Eigen and Schuster, 1979) further study of Kauffman introduced the term “*catalytic closure*” (Kauffman, 1986). It describes the condition in which “every molecule in the system either is supplied from the outside as ‘food’ or is itself synthesized by reactions catalyzed by molecular species within the autocatalytic system” (Kauffman, 1995, p. 49). He argued that once molecular diversity passes a critical threshold (in line with the von Neumann ‘threshold of rank’), such closure becomes highly probable, marking a phase transition in chemical networks toward autocatalytic closure.

$$\begin{array}{l} \text{Random symbolic}\;(\text{molecular})\;\text{replication}\to \\ \text{Critical threshold}\to \text{Catalytic closure} \end{array}$$

Further study of Kauffman links catalytic closure-to-thermodynamic work cycles (Kauffman, 1996), an idea that Morowitz suggested earlier: “in a steady state system (open systems), the flow of energy through the system from a source to a sink will lead to at least one cycle in the system” (Morowitz, 1968, p. 32). An important point in the introduction to thermodynamic cycles, in Kauffman’s view, is that work is needed to produce constraints, which in turn make it possible to transform energy into work, either to produce further constraints or to perform maintenance activities (Kauffman, 2000). This study, in which Rosen’s book (1991b) is briefly cited, links to a further extension in line with Montévil and Mossio (2015) (see below). Kauffman (1995) discussed autopoiesis in explicitly Kantian terms, insofar as each part exists both for and by means of the whole, while the whole exists both for and by means of the parts. However, for Maturana it goes beyond Kant in the sense that local molecular interactions in autopoiesis operate in the immediate locality without any reference to the totality they compose. As he noted, molecules are “blind to the consequences to which they give rise” (Maturana, 2002, p. 268). Under the scrutiny of Rosen’s theory, semantic closure and catalytic closure, although different—Pattee emphasizing symbolic rules in DNA and Kauffman eliminating the need for a central genome, share computability under the Turing machine paradigm. At least this is the conclusion of Rosen’s review of Kauffman’s *Origins of Order* (Rosen, 1994).

By this time, the principal authors were not only discussing their respective explanations of organizational closure among themselves, but secondary authors were also beginning to evaluate the relationships among those explanations. John Stewart, who was a close collaborator of Varela, put forward a thesis involving autopoiesis and the study of Pattee and Rosen: “a dynamic system is a living organism if and only if it produces and interprets semantic inscriptions” (Stewart, 2000, p. 161). Afterward, Zaretzky and Letelier (2002)^33^ for the first time showed an equivalence between autopoiesis and (M, R)-systems, allowing Letelier and co-workers to elaborate the concept of “*metabolic closure*” (Letelier et al., 2003, 2006, 2011). Metabolic closure explicitly introduces temporality into the analysis of organizational closure. This has reopened the issue of the dynamic realization of closure-to-efficient causation. Recently, a fruitful dynamical realization of metabolic closure has been analyzed (Yun-Cárcamo et al., 2020), although focusing on the stability rather than the closure itself (see footnote 9). All these explanations of closure had contributed to recharacterizing biological organization as “*closure of constraints*” (Montévil and Mossio, 2015). Constraints are defined as entities that act upon processes while remaining relatively conserved over the relevant time scales. They exhibit mutual dependence insofar as they both depend upon and contribute to maintaining one another. It is argued that the closure of constraints, similar to operational closure, leads to biological autonomy (Moreno and Mossio, 2015). This study has influenced Kauffman, who has proposed three forms of closure: “constraint closure; work task closure; and catalytic closure” (Kauffman, 2019, p. 30).

The significance of these developments is that the major explanatory theories of organizational closure have now converged on a common set of contemporary questions. The genealogy suggests that every closure theory attempts to solve the same problem: How can a living system become the cause of the conditions required for its own persistence? However, each explicative account apparently identifies a different “thing” that closes upon itself. The time issue however shifts the discussion from “What is closed?” to “What does closure do over time?” Recent work based on catalytic closure and assembly theory investigated the relationship between autocatalytic closure and open-ended evolution (OEE), providing numerical evidence that closure is not simply correlated with increasing diversification but may act as a causal driver of it (Faggian, 2025). That is, closure no longer appears only as a condition for persistence, but also as a possible source of evolutionary creativity. The diversity of biological lineages has been interpreted as different structural realizations of autopoiesis by Maturana and Varela (1987), Maturana and Mpodozis (1991), and Varela et al. (1991). Structural change was also theorized in the context of closure of constraints by Montévil and Mossio (2020) and Roli et al. (2025). Although catalytic closure is not the organizational closure found in autopoietic or (M, R)-systems (Chemero and Turvey, 2008)—since catalysts are not themselves generated through a fully closed network of efficient causes—the study of Faggian (2025) and Yun-Cárcamo et al. (2020) nevertheless reinforces the idea that closure introduces a distinctive temporal regime in which organization constrains and channels future possibilities. Here, viability theory (Aubin, 2009) may provide a useful mathematical framework for understanding how closure operates over time at a minimal level. Whereas Kauffman’s autocatalytic sets focus on the emergence of closure through mutually catalytic reactions, viability theory approaches the problem by asking under what conditions a system can remain within a set of admissible states over time. The central notion is the viability kernel: the set of states from which a system can continue evolving without violating the constraints required for its persistence. Rather than defining closure in terms of causal entailment, viability theory characterizes the conditions under which a system can maintain itself despite uncertainty, perturbations, and multiple possible trajectories. The emphasis shifts from the question “How are the causes of the system generated?” to “Under what conditions can the system continue to exist?” In this respect, viability theory complements rather than replaces autopoiesis, closure-to-efficient causation, or closure of constraints. However, within organizational closure, these conditions are not independent; rather, they are enacted through sense-making processes grounded in organizational dynamics. The emergence of meaning is traditionally the explanatory domain of biosemiotics, which argues that life is fundamentally a semiotic phenomenon and meaning is intrinsic to living organization (see, for example, the studies of Jesper Hoffmeyer or Claus Emmeche). The philosophical roots of biosemiotics are Peircean semiotics, von Uexküll’s *Umwelt* theory, and phenomenology. This makes biosemiotics naturally compatible with autopoiesis, enaction, and the Biology of Cognition. However, an alternative explanation argues that rather than meaning there are codes and that cells are not autopoietic systems, but rather systems capable of generating new organic codes (rule-based correspondences) and conserving the existing ones, that is, codepoietic systems (Barbieri, 2012, 2018). For Maturana and Varela, the notion of code is observer-dependent, and the relationship between Code Biology and Biosemiotics through Rosen’s Relational Biology is currently a subject of deep theoretical debate (Vega, 2018).

The relationship between organizational closure and meaning-making is important to understanding how AI, machine cognition, and large language models (LLMs) work. Contemporary AI requires acknowledging that transformer-based large language models are not merely static symbol manipulators in the classical sense. Recent architectures incorporate features that partially mimic some aspects historically associated with closure. In-context learning allows models to modify their behavior dynamically within a conversational episode without changing their parameters; agentic systems introduce recurrent action–observation loops through tool use and environmental feedback; persistent memory architectures enable information acquired in previous interactions to influence future behavior; and reinforcement learning from human feedback (RLHF) shapes stable behavioral dispositions that may appear as goal-directed tendencies. These developments move AI beyond purely feed-forward computation and introduce forms of operational circularity and adaptive regulation. Nevertheless, such architectures remain fundamentally dependent on externally specified objectives, training procedures, evaluation criteria, computational infrastructures, and energy flows. Their feedback loops do not regenerate the constraints that make those loops possible, nor do they fabricate the conditions of their own continued existence. What recurs are information flows, policy updates, and state transitions, not the mutual production of the efficient causes that constitute the system as an autonomous unity. Consequently, although contemporary LLMs may exhibit increasingly sophisticated forms of self-modification, memory integration, and adaptive behavior, these features are better understood as engineered approximations of closure rather than instances of organizational closure itself. In addition, recent neurobiologically grounded models of language (Martin, 2020; Murphy, 2025) support the claim that current LLMs lack endogenous semantic grounding, which is constitutively tied to biological/causal organization rather than to syntactic computation. LLMs—and, more generally, algorithmic procedures—therefore remain examples of computationally mediated heteronomy rather than organizationally closed systems capable of autopoiesis, constitutive autonomy, or intrinsic sense making.

Our aim here was to provide a genealogical conceptual analysis that determined the theoretical origins—spanning mathematical logic, first- and second-order cybernetics, general systems theory, thermodynamics, metabolic theory, relational biology, and the biology of cognition—of each of these theoretical explanations of organizational closure (Table 2). The genealogy suggests that the various notions are not independent explanatory principles but different formal approaches to explaining organizational closure as a biophysical complex phenomenon that Gregory Bateson (1978) called the “pattern which connects.” Organizational closure can also be interpreted as a biophysical counterpart of the “strange loop”—a theme reminiscent of Hofstadter’s analysis (Hofstadter, 1979). However, it is important to note that the strange loop is a formal and epistemic notion of self-reference across levels of description, derived from Gödelian incompleteness, Escher’s recursive drawings, and Bach’s recursive musical structures. Organizational closure, by contrast, concerns the causal closure of efficient causes in physical systems (living beings). Nevertheless, both approaches share a common ancestry in the problem of self-reference.

**Table 2:** Key genealogical contributions shaping the contemporary characterization of organizational closure.

Domain	Key innovation/type of closure introduced	Contribution to the modern characterization of organizational closure
Mathematical logic	Logical paradoxes; self-reference; incompleteness; limits of syntactic closure	Shows that meaning and entailment cannot be reduced to computation (syntax); motivates the need for endogenous semantic organization.
Cybernetics (first/s order)	Circular causality; feedback closure; closure to information; cyclic closure (Piaget)	Introduces the idea of circular organization but reveals the limits of feedback as genuine closure; prepares the shift toward operational autonomy.
General systems theory and theoretical biology	Open systems; self-maintaining dynamics; distinction between order and organization	Establishes that biological autonomy exceeds thermodynamic order; highlights the need for constraint-based organization.
Biology of cognition/autopoiesis	Operational closure; constitutive autonomy; self-production	Characterize the materiality realization of closure in living systems; link organizational autonomy with cognition.
Relational Biology/(M, R)-systems	Closure-to-efficient causation; entailment structures	Provides the first formal model of an organizationally closed system; defines self-fabrication as internal causal entailment of efficient cause.
Catalytic closure; semantic closure; metabolic closure; closure of constraints	Supplies minimal material architectures capable of proto-closure	Bridges the organization closure with prebiotic and biochemical realizations.

The genealogical approach presented here provides a set of criteria for classifying the various explanations of organizational closure (Table 3). Furthermore, this genealogical approach reveals a common thread that provides indications of their equivalences, that is, whether and to what extent they are similar and dissimilar, and what their relations are, both conceptual and mathematical, between them and other proposals.^34^ Recent study has explored the equivalences of the various explanations of organizational closure through graph-theoretical approaches (Brown and Vittadello, 2025), yet often without a sufficiently clear account of what it actually is, or of its historical and explanatory diversity. A robust understanding of closure—and the methods used to model it—must ultimately rely on the ability to detect when closure occurs, as emphasized by Chemero and Turvey (2008).^35^

**Table 3:** A set of criteria for classifying the various explanations of organizational closure.

Criterion	Question	Examples
Unit of closure	What is closed?	Information (Ashby), operations (Maturana), efficient causes (Rosen), constraints (Mossio), and formal catalysts (Kauffman)
Type of circularity	What returns to itself?	Bits, signals, operations, causal roles, constraints, and semantic functions
Causal regime	What kind of causation is emphasized?	Efficient, formal, final, informational, thermodynamic, and a set of them
Material realization	Is closure abstract or embodied?	Logical systems, neural systems, immune systems, cell systems, metacells, and Gaian-ecosystems
Relation to computability	Is closure computable?	Strongly computable (Ashby, Kauffman), partially computable (Pattee), non-computable (Rosen, Maturana, and Varela)
Role of semantics	Is meaning intrinsic or observer-assigned?	External (Shannon), hybrid (Pattee), intrinsic (Maturana, Varela, and Rosen)
Source of autonomy	What generates autonomy?	Feedback, self-organization, self-production, and causal entailment
Biological scope	What phenomena are explained?	Unitary individuation, metabolism, regulation, cognition, evolution, and life–mind continuity

Finally, the genealogical reconstruction developed here enables us to distill organizational closure into four complementary dimensions: its physical-causal structure, its epistemological implications, its explanatory value for biological organization and lived phenomenology, and its relevance for future research in synthetic biology and cognitive science.

- Physical-causal definition

At its core, organizational closure is the circular entailment of efficient causes that fabricate and regenerate one another while maintaining their own boundary conditions. Unlike transient self-organizing processes that persist only while external gradients are maintained, organizationally closed systems actively conserve and re-establish the constraints that make their own continuation possible. They remain open to external flows of matter and energy; however, the causal roles required for their persistence are internally generated and continuously reconstituted.

- Epistemological implications

Syntactic or state-determined models cannot fully capture this causal regime. As organizational closure requires the endogenous entailment of causal roles—and thereby entails a dynamic interplay among efficient, material, and final causes—it exceeds the descriptive resources of Newtonian mechanics and computation in the Turing sense. This marks a decisive break with both Hilbert’s program and the strong interpretation of the Church thesis. Living systems instantiate causal architectures that cannot be exhaustively characterized in algorithmic terms, and their semantic, autonomous, and anticipatory properties cannot be derived from externally imposed rules or fixed transition structures (Rosen, 1991b; Varela, 2000; Louie, 2009).

- Theoretical value

Organizational closure provides a principled criterion that distinguishes living from non-living systems. It provides a proposed necessary and sufficient criterion for distinguishing living from non-living systems. It generalizes Bernard’s “milieu intérieur” and clarifies why physical order, thermodynamic openness, or informational feedback are insufficient to account for biological organization (Rosen, 1991b; Varela, 2000; Louie, 2009). Organizational closure should be considered whenever we attempt to understand, reproduce, or intervene in biological phenomena—from minimal artificial cells and wetware systems to ecological and perhaps Gaian-scale processes. From this perspective, organizational closure can be understood as the causal architecture underlying what Bateson called “the pattern which connects,” grounding the continuity between life, mind, and organized agency.

- Future directions: relevance for synthetic and cognitive research

The genealogical clarification of organizational closure developed in this article also opens several avenues for the future study. First, it provides a conceptual foundation for contemporary attempts to construct minimal forms of biological autonomy in wetware systems, such as protocellular models and chemically embodied networks capable of sustaining endogenous causal roles. These synthetic platforms allow the exploration of proto-forms of closure—such as catalytic and metabolic loops—that may arise under prebiotic conditions, while also testing the circumstances under which such loops could collectively generate the internal entailment of efficient causes characteristic of organizational closure. In this sense, the notion of closure offers a principled framework for interpreting experimental advances in minimal life, synthetic cells, and chemically embodied cognition. Recent theoretical and experimental works on minimal wetware architectures have already begun to explore these possibilities, suggesting how chemically embodied systems might instantiate early forms of causal self-maintenance (Stano and Damiano, 2023; Rubin et al., 2024, 2025; Damiano et al., 2026).

Second, the account developed here bears directly on current debates in cognitive science and AI. As large-scale computational architectures such as transformer-based language models display increasingly sophisticated abilities, questions concerning the nature of meaning, understanding, and explanation have re-emerged with renewed urgency. The analysis of organizational closure clarifies why such systems, despite their impressive performance, lack the endogenous causal organization required for biological autonomy: their functional architecture remains heteronomously specified through externally defined objectives, training procedures, and constraints, rather than being internally generated. Organizational closure therefore provides a distinctive criterion for assessing both the capacities and the limits of computational simulations of cognition and for articulating the continuity among, mind, and organized agency.

Finally, this framework suggests a broader research agenda. It invites investigation into how different forms of causal organization—computational, dynamical, metabolic, and ecological (Stano and Damiano, 2023; Rubin et al., 2025)—can be systematically compared, and under what conditions artificial systems might approach, approximate, or model aspects of organizational closure without instantiating it. Such a program would contribute not only to synthetic biology and artificial life but also to the conceptual foundations of cognitive science, grounding future inquiries into autonomy and sense making from ambiguity and logical paradox.

## Footnotes

1 S: This statement (S) is false. If (S) is true, then ‘This statement is false’ is true. Therefore, (S) must be false. The hypothesis that (S) is true leads to the conclusion that (S) is false, a contradiction; If (S) is false, then ‘This statement is false’ is false. Therefore, (S) must be true. The hypothesis that (S) is false leads to the conclusion that (S) is true, another contradiction; Either way, (S) is both true and false, which is a paradox.

2 The arrows summarize conceptual trajectories discussed in the argument and indicate relations of theoretical interpretation rather than causal mechanisms.

3 In the late 1950s, cybernetic ideas circulated widely across Europe through the International Congresses on Cybernetics in Namur (held in 1956 and 1958). Participants included figures such as Beer, Minsky, Pask, and von Foerster, whose encounter at these meetings proved influential. Around the same period (1958), the Dartmouth Conference on “The mechanization of thought processes” —organized by Minsky and Shannon at the National Physical Laboratory (Davies, 1959) — helped consolidate the emerging symbolic approach to cognition that would later define artificial intelligence. McCulloch, Ashby, Beer, Rosenblatt, and Pask were among attendees.

4 The self-reproducing automaton is a finite-state system like Turing machines that displays pattern generation and self-organization defined by externally imposed transition rules. Their efficient causal structures execute preprogramed instructions and thus remain heteronomous: they are open-to-efficient causation in the sense that their organization is not self-produced.

5 In 1960, three major symposia on self-organizing systems were held: two organized by Yovits and Cameron (1960) and one by Zopf and von Foerster (1962). The latter brought together key figures of cybernetics and systems theory—including Bertalanffy, Ashby, McCulloch, Beer, Pask, Rosenblatt, Rappaport, Sperry, and Hayek—and contributed significantly to the circulation of ideas on self-organization within the cybernetic community. In this context, Ashby presented his influential study on self-organizing systems (Ashby, 1962a), which later resonated with developments in the Biological Computer Laboratory (BCL) and in Maturana’s formulation of state-determined systems Maturana (1975).

6 For a comprehensive interpretation of Schrödinger’s *What is Life?* that is not aligned with the traditional interpretation of molecular biology dogma and its “sequence hypothesis” determinism, see mainly chapter 1 of Rosen (Rosen, 1999) and Kauffman (2020).

7 Hans Driesch was a former student of August Weismann at the University of Fribourg and of Ernst Haeckel at the University of Jena in 1886 and 1887, respectively.

8 The concept is intimately connected with the distinction between matter and form, or the potential and the actual.

9 For a recent approach to how organizational closure in terms of constraints fosters biological stability, as well as its interaction with variation, see Mossio et al. (2016).

10 Bertalanffy’s work would be well understood by Ashby, especially in the development of state-determined systems. Still, above all, the one who would better understand the study and the implications of von Bertalanffy’s biology will be Rosen (see footnote 29).

11 In 1954, von Bertalanffy co-founded the Society for General Systems Research together with scholars such as Rapoport, Gerard, and Boulding. The society, later renamed the *International Society for Systems Science (ISSS)*, gathered key figures in systems thinking, including Ashby, Rosen, Klir, and Prigogine, and played an important role in institutionalizing the study of complex and open systems.

12 During the 1960s, several meetings helped consolidate a research agenda centered on biological organization, development (epigenesis), heredity (memory and fidelity), theory of evolution beyond neo-Darwinism, and the thermodynamic foundations of life from the emerging field of theoretical biology (Waddington, 1968). These included the lectures on theoretical and mathematical biology organized by Morowitz at Yale (Waterman et al., 1965), Waddington’s Bellagio symposia on theoretical biology (1966–1967), and Danielli’s NASA-sponsored Summer Colloquium on Theoretical Biology in Colorado, CO (Danielli, 1969). The Bellagio symposia brought together Goodwin, Howard Pattee, Arbib, Rossler, Elsasser, Thom, Kauffman, Conrad, among others. In contrast, the Colorado Colloquium brought Rosen, Ashby, Pattee, Conrad, Kauffman, Goodwin, and Newman, where Ashby and Rosen interacted intensively over a 6-week period (Rosen, 1985e).

13 Von Foerster founded the BCL in 1958 following a sabbatical year with Warren McCulloch and Arturo Rosenblueth. This occurred 10 years after he published *Memory: A Quantum Mechanical Treatise* (von Foerster, 1948), which was reviewed by Erwin Schrödinger, who “did not believe a word of it, but he could not find any error in the mathematics” (von Foerster, 2003). However, it served as his vehicle to editing the Macy Conferences (von Foerster, 1952) via McCulloch’s invitation. McCulloch subsequently recommended von Foerster for a professorship at the University of Illinois in 1951. He directed the BCL until his retirement in 1975. The BCL hosted Ashby, Beer, Löfgren, von Glasersfeld, Maturana, Varela, and Pask, among others. The latter three were frequent visitors during the 1960s and 1970s. Maturana’s final visit to the BCL took place in early 1974, 1 year before von Foerster retired and the BCL ceased operations.

14 In a recap of how Maturana arrived at these ideas, he recounts that in the 1960s he considered that: “*…scientists such as Ludwing von Bertalanffy, adopting a systemic approach in the quest for understanding living beings as totalities, spoke of an organismic vision, apparently thinking that to understand living beings it was central to attend to their constitution as open systems and to the way they processed energy. I, on the other hand, thought that the key to explaining and understanding living beings was acknowledging their condition as discrete autonomous beings”* (Maturana et al., 2011, p. 584).

15 A recent, albeit computational, framework called free energy minimization (also known as the Free Energy Principle [FEP]) rescues these action–perception sensorimotor loops as dynamics of active states and sensory states for active inference within the perceptual system (Friston, 2013). The FEP can be interpreted as a formal systems approach to this relational domain of autopoiesis, describing perception and action through sensory and active states, respectively, whereby internal states infer the causes of sensory perturbations to maintain viability and reduce uncertainty. Although several authors argue that a fully enactive interpretation of structural coupling requires autonomous modifications of the organism’s dynamical laws and state spaces (Di Paolo et al., 2022), the FEP nevertheless constitutes a tractable framework for investigating cognition in a spirit compatible with action-perception loops of the Biology of Cognition (Kiverstein et al. 2022; Rubin 2023).

16 Maturana was not a mathematician and never explicitly engaged with Gödel’s work. It is highly probable that his dissatisfaction with the concept of self-reference represented neither a logical paradox nor an issue of impredicativity in the formal mathematical sense, but rather a tension in part–whole relationships that he found difficult to reconcile with autopoiesis. This was partly because: “talking about living beings as self-referring systems was not satisfactory…because the notion of ‘self-reference’ subordinates the dynamics of the components of a system to the totality that results from their operation, which was the very thing that I wanted to avoid when I talked about the local relations of the components of a living being, showing that the living being in its existence as a totality was a spontaneous result of that manner of operation. Indeed I was aware that talking about living systems in terms of self-reference obscured the fact that I had not yet identified the operational dynamics that made a living being a self-referring entity*”* (Maturana et al., 2011).

17 Varela became a student of Maturana in 1966 when he knocked on the door of Maturana’s experimental epistemology laboratory at the Universidad de Chile, asking to study “the psychics of the universe” (Maturana et al., 2011). He subsequently pursued his Ph.D. in insect vision (1967–1970) at Harvard University, where he met von Foerster in 1968 and became a frequent visitor to the BCL (von Foerster et al., 1985). By the end of 1969, Varela encountered Piaget’s (1967). Varela returned to Chile in 1970, and by the end of that year, he and Maturana began developing the autopoietic characterization of living systems. By 1972, Varela brought a fresh copy of the manuscript to a meeting organized by Ivan Illich in Cuernavaca, Mexico, which Pask and von Foerster attended. In their resulting book, however, there is no explicit reference to “closure” or “closed organization,” but rather to a causal, “closed domain of relations” of molecular production. Furthermore, for Maturana and Varela, regulation (and self-regulation in particular—a central piece of cybernetic terminology at the time) depends entirely on the observer and does not belong to the intrinsic organization of the living. Autopoiesis was fully discussed in July 1973 during a series of seminars titled *The Cybernetics of Cognitive Processes* in Santiago de Chile, delivered by Stafford Beer, Humberto Maturana, and Heinz von Foerster.

18 Both Maturana and Varela independently later came to the conclusion that self-reproducing automata were not the correct characterization of organizational closure. While Varela not only published his commented Calculus of Self-Reference (Howe and von Foerster, 1975; Varela, 1975), but indicated that the living *“*characterization in terms of a von Neumann machine, the input–output paradigm, etc., were not appropriate*”* (Varela et al., 1985, pg. 291), Maturana concluded that *“*I do not consider what take place in the operation of von Neumann’s machine as a model of cellular reproduction. In fact, I think that von Neumann superbly realized in his machine a then current description of a biologically mistaken characterization of the processes that take place in cellular division*”* (Maturana, 1980, p. 324). Still, apparently, their conclusion was not connected to Ashby’s agreement with Rosen’s argument about the inconsistency of self-reproducing automata as a model of organizational closure (Ashby, 1962b). As a matter of fact, nowadays self-reproducing automata remain the current model of living systems from molecular biology and physical perspectives (Brenner, 2012; Walker and Davies, 2013).

19 In this study, published in *The Conference Proceedings of the First International Symposium on Biosimulation* (which took place in Locarno, Switzerland, in 1960), Ashby (1962b) explicitly acknowledges the influence of Rosen’s study (Rosen, 1959). Among the attendees and contributors to the conference were Donald MacKay, Gordon Pask, Warren McCulloch, and Heinz von Foerster; the latter contributed a study titled *Circuitry of Clues to Platonic Ideation*—a text that deeply influenced Varela when he began working in Maturana’s lab (Varela, 2011).

20 Löfgren was perhaps the member of the BCL who knew Rosen’s study best, representing the point of intersection between the work produced by the BCL and the Committee on Mathematical Biology (which would later drive the founding of the CTB) at the University of Chicago. He also wrote a review of Rosen’s major treatise on dynamical system theory (Rosen, 1970; Löfgren, 1974). Other members of the BCL and CTB were well acquainted as well: von Foerster and Ashby were close friends, and Ashby met both Maturana and Rosen personally. Howard Pattee worked closely with Rosen and met Varela personally, while Ludwig von Bertalanffy worked with Rosen and also met von Foerster.

21 When Stengers asked von Foerster about the origin of the idea of closure, he said: “We were all in very close cooperation, and we were not concerned with who thought of what, but only with understanding it. We cannot know who had the idea, who invented this or that. In the laboratory, whoever had an idea told everyone about it; it was the mutual discussions that made it possible to clarify, to bring together people who knew different things. Certainly, Maturana was one of those who insisted on this type of point of view, and who, of course, pushed the idea of closure very vigorously, particularly in the case of perception” (von Foerster et al., 1985). Pask mentioned that organizational closure was a current linguistic coin in his own activity: “The boundaries of the system, far from being pre-fabricated, are created by the activities of the system. This is a prescient notion of autopoiesis (as developed by Maturana and Varela), or organizational closure, as we call it at Brunel University and in my own laboratory, System Research” (Pask, 1996). On the other hand, the first published appearance of the term “organizational closure” belongs to Varela (1979). Varela recalled: “at the time (around 1969), I had also discovered Piaget, for me the only European who was going in the same direction, although for me in a rather “spaghetti” way…the first was the explicit formulation of the idea that, whatever the living organization, and the knowledge of that organization, are inseparable from its quality of being autonomous…And that produced the book on autopoiesis, which led me to become interested in circular causality…indeed, what I discovered or rediscovered at that time was not discontinuous with the BCL’s questions. In addition, the BCL people liked these ideas. However, I did not find them there. It was something I had to redo. According to Heinz himself, we were adding something that wasn’t there; it wasn’t simply saying the same thing with other words*”* (Varela et al., 1985).

22 On the front page of Rosen’s book “*Fundamentals of Measurement and Representation of Natural Systems*,” an alternative working title for Varela’s forthcoming *Principles of Biological Autonomy* appears as *Whole Systems: The Cybernetics and Epistemology of Self-Reference* (Rosen, 1978c). Moreover, before settling on the final title, Varela opted for alternatives such as *Autonomy and Cognition: On the Circularities of Natural Systems and their Cognitive Processes* or *Patterns of Autonomy*, cited in the bibliographies of (Kauffman and Varela, 1980) and Varela (1978), respectively. These evolving titles reflect the genealogy and synthesis of organizational closure from self-reference (mathematics), whole systems (GST), circularity (cybernetics), natural systems (relational biology), and autonomy (biology of cognition).

23 A timely new edition of *Principles of Biological Autonomy*, revised and annotated by Ezequiel Di Paolo and Evan Thompson, with a foreword by Amy Cohen Varela (Varela, 2025), and a new Italian edition of *The Tree of Knowledge* (Maturana and Varela, 2024), featuring an essay by Mauro Ceruti and Luisa Damiano, have recently been published.

24 Founded and led by William Irwin Thompson, Lindisfarne brought together figures such as James Lovelock, Lynn Margulis, Henri Atlan, Carl Sagan, William Irwin Thompson, Ralph Abraham, David Abram, Humberto Maturana, Francisco Varela, Louis Kauffman, and Gregory Bateson, alongside parallel dissemination through outlets such as *Whole Earth* and *CoEvolution Quarterly*.

25 Rashevsky’s institutional impact was significant in many ways. The University of Chicago established a Section on Mathematical Biophysics (later the Committee on Mathematical Biology) under his leadership. This effort was instrumental in founding the Society for Mathematical Biology and heavily influenced James Danielli in establishing the Center of Theoretical Biology (CTB) at SUNY Buffalo and the *Journal of Theoretical Biology* in the early 1960s. Rashevsky maintained active correspondence with Vladimir Vernadsky (who coined the term *biosphere*), G. Evelyn Hutchinson (author of *The Fitness of the Environment* frameworks), and Norbert Wiener in the mid-to-late 1940s, with Warren Weaver during 1948–1954, with Warren McCulloch during 1952–1956, and with Heinz von Foerster in 1963. See the Rashevsky Archives, University of Chicago.

26 The arguments Rosen deploys in this study, beyond the identification of the enzyme’s active site as the catalyst, bear a striking structural similarity to the arguments published independently by Maturana and Varela in the same year (Varela and Maturana, 1972).

27 The formalization of Aristotelian causality within the language of Category Theory was well received by René Thom in his reviews of Rosen’s study (Thom, 1986). Thom himself adopted Aristotelian causality for the study of organisms (Thom, 1988), and both authors contributed to an entire volume dedicated to the topic; see Casti and Karlqvist (1989). Rosen and Thom influenced each other deeply, sharing a skeptical view of classical Newtonian physics in general and of Prigogine’s study in particular.

28 The theoretical foundations of molecular biology and current physical perspectives on life could be fundamentally challenged on the basis of the formal arguments established in this study. (see footnote 18).

29 Final causation as anticipation distinguishes (M,R)-systems from autopoiesis as it was initially postulated, and it marks a point of departure between Maturana and Varela’s later perspectives. Maturana argued that organisms are structurally determined systems: their behavior depends entirely on their current structural configuration—the result of a history of structural coupling—rather than on externally specified or future goals (Maturana and Poerksen, 2004). Maturana moved toward structurally determined systems (Maturana, 1980) by adopting Ashby’s state-determined systems, which was itself influenced by Bertalanffy’s study (Maturana, 1975). Rosen understood that mechanisms do not admit final causes because mechanisms are state-determined systems (Rosen, 1979, 1985e): “Ashby’s general approach to biological problems was not reductionistic, but it was Cartesian and Newtonian. That is to say, Ashby was a mechanist. Indeed, in many ways he represented a kind of culmination of the mechanistic approach” (Rosen, 1985e). Varela later identified this mechanistic underpinning as a distinct limitation of the early formulations of autopoiesis (Varela, 1981). He subsequently proposed moving beyond the mere “as-if” character of internal purposes immanent to the living to grasp “immanent teleology as a truly biological feature” (Weber and Varela, 2002), though this lacked a complete formalization of its organizational basis. Within relational biology, final causation reappears on rigorous formal grounds not as an external purpose but as an immanent property of closure-to-efficient causation, defined in anticipatory terms (Rosen, 1985a, 1991b; Louie, 2012). Recent developments argue for a structural integration of the theoretical legacies of anticipatory systems and structurally determined systems, focusing on how organisms select an environmental space of admissible interactions that trigger ontogenetic and evolutionary changes (Rubin, 2017, 2022). See Astorga and Letelier (2026) for a reconciliation of structural coupling with anticipation.

30 George Klir was a key figure in spreading the study of members of the BCL and CTB, and thus of Maturana, Varela, Rosen, Pattee, among others. Klir was also the founding editor and editor-in-chief of the *International Journal of General Systems* from 1974 to 2014.

31 This journal was founded in 1978 by James Danielli and later joined by Eugene Yates, Rene Thom, and Rosen, who also served as co-editor from 1985 to 1989. Pattee and Elsasser also published there.

32 McCulloch supported Kauffman’s early study, which Kauffman developed at the Department of Theoretical Biology of the University of Chicago and the Committee on Mathematical Biology (Kauffman, 1969). Kauffman visited McCulloch for 3 months in 1967, and McCulloch invited him to his house (Kauffman, 1990). The study that Kauffman undertook under McCulloch’s support was to characterize the typical behaviors of gene nets of binary devices, subject only to constraints on the number of “genes” in a net, N, and the number of genes regulating any given gene’s activity, K. The so-called NK model.

33 Zaretzky was a student of Carlos Leguizamon, who was an Argentenean student of Rosen during the 70s at the CTB and one of the pioneers in bringing theoretical biology to Latin America. Letelier was a student of Maturana and Varela.

34 Notable examples of these principles include the hypercycle (Eigen and Schuster, 1979), the chemoton (Gánti, 2003), and the autogen—a unitary system exhibiting a constraint coupling between self-organization (autocatalysis) and structural self-assembly—which suggests that such constraint coupling is inherently teleodynamic (Deacon, 2021). For instance, Kauffman has argued that autocatalytic sets closely resemble the autogen (Kauffman, 2021), align with autopoiesis, and exemplify a distinct “closure of constraints” (Kauffman, 2019). By contrast, Pattee has questioned the theoretical plausibility of the autogen as a viable model of living systems (Pattee, 2021).

35 This ontological and epistemological issue becomes particularly salient in contemporary attempts to design artificial autonomous systems. A close examination of these systems reveals that a degree of heteronomy is typically embedded within the design process itself, as human designers inevitably introduce external constraints and normative choices that fundamentally shape the system’s organization. Furthermore, questions whether true organizational closure has been achieved frequently re-emerge when the analysis is shifted to descriptive levels other than those initially specified by the model. The physicochemical domain may offer a promising empirical starting point for resolving these ambiguities, given that molecular structures and thermodynamic interactions already embody primitive forms of constrained autonomy. Current engineering efforts to construct artificial life-like frameworks or chemically embodied cognitive systems benefit directly from these intrinsic properties and would gain further clarity from a deeper engagement with the theoretical and genealogical issues discussed herein (Deplazes-Zemp, 2016; Bich and Frick, 2018; Stano and Damiano, 2023).
